# A Perspective on the Success and Failure of BCG

**DOI:** 10.3389/fimmu.2021.778028

**Published:** 2021-12-14

**Authors:** Pawan Kumar

**Affiliations:** Department of Preventive Oncology, Dr. B. R. Ambedkar Institute Rotary Cancer Hospital, All India Institute of Medical Sciences, New Delhi, India

**Keywords:** tuberculosis, BCG, vaccine efficacy, immune response, environmental mycobacteria, heterogeneity, geographical latitude

## Abstract

TB continues to be one of the major public health threats. BCG is the only available vaccine against TB and confers significant protection against the childhood disease. However, the protective efficacy of BCG against adult pulmonary TB, which represents a larger burden of disease, is highly variable. It has been suggested that prior exposure to environmental mycobacteria (EMb) mitigates the anti-TB efficacy of BCG by blocking its duplication or masking its immunogenicity. However, its effectiveness against childhood TB and failure of repeated administration to provide additional benefit against pulmonary TB, suggest of some other mechanisms for the variable efficacy of BCG against the pulmonary disease. Importantly, TB is a heterogeneous disease occurring in different forms and having distinct mechanisms of pathogenesis. While inability of the immune system to contain the bacilli is responsible for TB pathogenesis in infants, an aggravated immune response to *Mtb* has been blamed for the development of adult pulmonary TB. Available data suggest that EMb play a key role in heightening the immune response against *Mtb*. In this article, differential efficacy of BCG against childhood and adult TB is explained by taking into account the heterogeneity of TB, mechanisms of TB pathogenesis, and the effect of EMb on anti-*Mtb* immunity. It is believed that a refined understanding of the success and failure of BCG will help in the development of effective anti-TB vaccines.

## Introduction

Tuberculosis (TB) continues to be one of the major public health threats, accounting for approximately 1.5 million deaths per year across the globe. BCG (Bacillus Calmette-Guerin), which was developed nearly 100 years ago, is the only available vaccine against TB. Despite conferring significant protection against childhood manifestations of TB, protective efficacy of BCG against adult pulmonary TB is highly variable (1, 2). Lowest efficacy of BCG against adult pulmonary TB has been observed in the tropics, which account for the major burden of the disease. Why BCG confers significant protection against childhood TB but fails against the adult pulmonary disease remains an open question.

*Mycobacterium tuberculosis* (*Mtb*), the causative agent of TB, shares an intricate relationship with the host immune system and leads to different clinical outcomes. Therefore, TB is considered a heterogeneous disease, occurring in different patterns and presentations and having distinct mechanisms of pathogenesis (3). In young children, *Mtb* infection leads to the primary disease commonly affecting extra-pulmonary sites. On the other hand, TB develops after reactivation of latent infection and preferentially affects lung tissue in most immunocompetent adults. Among various factors involved in defining the efficacy of BCG and the outcome of *Mtb* infection, exposure to environmental mycobacteria (EMb) has been shown to play a particularly important role (1).

In the present manuscript, I shed light on the variable efficacy of BCG and explained it by taking into account the heterogeneity of TB, mechanisms of TB pathogenesis, and the effect of EMb on anti-*Mtb* immunity. It is believed that a refined understanding of the success and failure of BCG will help in the development of effective anti-TB vaccines.

## BCG Vaccine

BCG was derived by French researchers Albert Calmette and Camille Guerin by *in vitro* passaging *M. bovis* for nearly 13 years. Clinical studies with BCG took place in France and Belgium in the 1920s and demonstrated its efficacy against childhood TB (4). As the effectiveness of BCG against childhood TB was observed in these and other European countries, World Health Organization (WHO) recommended expansion of the BCG vaccination program to the TB-endemic countries (1).

Despite more than 3 billion people receiving BCG, TB continues to be a devastating disease worldwide. A major drawback of BCG is its variable efficacy (ranging from nil to 80%) against adult TB. Palmer and colleagues were first to recognize that BCG is more effective against pulmonary TB at higher latitudes (1). Various factors including strain variations and poor cold-chain maintenance were suggested to be responsible for the variable efficacy of BCG against the adult disease. However, as the same BCG strains exhibited higher anti-TB efficacy in other countries, and were effective against leprosy in areas of their poor anti-TB efficacy, role of these factors in variable efficacy of BCG against adult TB was proved to be unfounded (5).

Currently, two hypotheses (masking hypothesis and blocking hypothesis) are considered most pertinent for explaining the failure of BCG against pulmonary TB. Masking hypothesis was based on the work by Palmer and colleagues, who observed in large-scale experiments in guinea pigs that immunization with EMb induces an appreciable level of protective immunity against *Mtb* and that the effects of subsequent BCG vaccination in these animals are markedly reduced (6). It was deduced that EMb, while imparting certain degree of protective immunity against *Mtb*, mask the efficacy of BCG to confer protection against the TB bacilli (6). Blocking hypothesis was based on the work by Anderson and colleagues, who demonstrated with a mouse model that prior exposure to EMb induces potent antimycobacterial immune responses, which block the duplication of BCG and thereby abrogate its efficacy to induce protective immunity against subsequent *Mtb* infection (7). As BCG is a live attenuated vaccine, its duplication in immunized people was considered as a precondition for induction of effective immunity against *Mtb*.

In either case, augmenting antimycobacterial immune response with booster doses of BCG could have been a potent strategy to enhance the anti-TB efficacy of BCG. However, repeated BCG administration has been found to not confer additional protection against pulmonary TB (although protection against leprosy is improved) (8, 9). Also, it is unlikely for the infants to receive significant EMb exposure before BCG vaccination, for they are immunized soon after birth. Moreover, the above hypotheses are based on animal studies, which have yielded contrasting result, with their applicability in humans difficult to verify (10–12). Therefore, why BCG fails to confer significant protection against adult pulmonary TB needs to be revisited.

## Heterogeneity of TB

TB is a heterogeneous disease occurring in different patterns and presentations (13). Two distinct presentations of TB can be seen in infants and immunocompetent adults (14, 15). In infants and young children, *Mtb* infection leads to primary disease with a high mortality rate. This form of disease commonly affects extra-pulmonary sites and in severe cases, can occur in disseminated form (miliary TB) (15). On the contrary, initial *Mtb* infection in immunocompetent adults is mostly contained as latent TB (LTB). This state of asymptomatic infection may persist lifelong in most but 5-10% of people, who would develop active disease during their lifetime (3). Active TB in immunocompetent adults preferentially affects lung tissue.

The primary and extra-pulmonary nature of TB in infants/young children is suggestive of their incompetence to contain *Mtb.* In keeping with this, immunological milieu in young children has been shown to be skewed towards the T_H_2 side, with dampened T_H_1-type of immunity and inflammatory pathways (16). Moreover, CD4^+^ T cells in young children are recent thymic emigrants with defective functionality and differentiation bias towards T_H_2 effector cells (17). That is why most children would respond poorly to mycobacterial antigens during tuberculin skin testing (TST) and IFN-γ release assay (IGRA) (15). On the other hand, immune system is effective against *Mtb* in most adults. These people mount a robust T_H_1 type of immune response to *Mtb*, resulting in sequestration of infected macrophages into lung granulomas and containment of infection as LTB (18). Paradoxically, host immune system has also been implicated in the reactivation of latent infection into active TB in immunocompetent adults (19).

## Protective *Versus* Pathological Immunity During *Mtb* Infection

Host response to *Mtb* begins with its recognition by resident lung macrophages which, along with dendritic cells, induce adaptive immune responses to bacilli. T_H_1-polarized CD4^+^ T cells are the key orchestrators of protective immunity against *Mtb* (18). Critical role of these cells in protection against *Mtb* is demonstrated by increased risk of TB in CD4^+^ T cell-lymphopenic HIV/AIDS patients. Similarly, CD4^+^ T cell depletion from animal models has been shown to increase the susceptibility to mycobacterial disease. Antimycobacterial functions of CD4^+^ T cells are partly mediated by TNF-α and IFN-γ, which induce bactericidal mechanisms in infected macrophages and facilitate their sequestration in the granulomas (3). Other cell types including CD8^+^ T cells, NK, and NKT cells have also been shown to contribute towards host resistance to *Mtb* (3).

LTB, which follows initial *Mtb* infection in most adults, is considered a state of protection against *Mtb* (20). It is characterized by a moderate anti-*Mtb* immune response in comparison to active pulmonary TB, wherein an aggravated antimycobacterial immunity is commonly observed (19). Heightened IFN-γ^+^CD4^+^ T-cell response to mycobacterial antigens, as evidenced by tuberculin reaction, IGRA, and *in vitro* assays, occurs frequently in active TB patients and is an important parameter distinguishing active TB from latent infection (21–24). Indeed, increased IFN-γ levels in different tissues is one of the most common and consistent observations during active TB (3). Importantly, IFN-γ levels directly correlate with TB severity and subside with its successful treatment, suggesting that mycobacterial burden could also contribute towards the aggravation of anti-*Mtb* immune responses (3). It is worth mentioning that host genetics also plays an important role in tuberculin sensitivity and some people may not exhibit tuberculin reactivity despite active disease (25, 26). Interestingly, these people have been shown to exhibit fewer symptoms but a more advanced disease (25).

TB-associated immune reconstitution inflammatory syndrome (TB-IRIS), which develops with the reactivation of asymptomatic *Mtb* infection in a subset of antiretroviral therapy (ART)-treated HIV-infected people, provides direct evidence for the pathological role of aggravated immunity during *Mtb* infection. ART-mediated decline in viral load results in the over-expansion of *Mtb*-specific CD4^+^ T cells in HIV and *Mtb*-coinfected patients (27). Lower baseline CD4^+^ T-cell count and rapid increase in these cells number with ART are the major risk factors for the development of TB-IRIS (28). Importantly, ART-treated people who are destined to develop TB-IRIS exhibit a more strong T_H_1-type of CD4^+^ T-cell response to *Mtb* antigens (27). Similar results have been obtained from animal studies, wherein the human condition was mimicked by adoptively transferring CD4^+^ T cells in *M. avium*-infected, TCRα^−/−^ mice (29). After receiving CD4^+^ T cells, these animals developed an aggravated immune response to the bacilli, exhibited wasting, lost weight, and eventually died of exacerbated immunity (29). Interestingly, development of immune reconstitution disease in these animals was mediated by hyperactive IFN-γ^+^CD4^+^ T cells.

The course of *Mtb* infection in PD-1^−/−^ mice also implicates aggravated CD4^+^ T-cell response in TB pathogenesis. PD-1 is a cell-surface receptor involved in the negative regulation of T-cell responses, and its deficiency results in significantly increased susceptibility to mycobacterial diseases, compared with wild-type mice (30, 31). Mechanistic studies have shown that PD-1^−/−^ mice mount a hyperactive CD4^+^ T-cell responses to mycobacteria, which drive mycobacterial pathogenesis in these animals (32). In agreement with animal studies, PD-1 blockade in cancer patients (a type of immunotherapy) has resulted in multiple cases of TB (33). It is noteworthy that PD-1 blockade-mediated TB development is associated with increased frequency of *Mtb*-specific IFN-γ^+^CD4^+^ T cells (33).

Significantly higher risk of TB in the immunocompetent adults cured of its previous episode also implicates aggravated CD4^+^ T-cell responses in TB development (34). *Mtb*-specific CD4^+^ T-cell responses are elevated during active TB, and a proportion of these cells can persist as memory cells after successful treatment (35, 36). It has been estimated that ~70% of people cured of pulmonary TB exhibit positive tuberculin reaction and IGRA up to 30 years after treatment (37). With *Mtb* reinfection, these hosts are more likely to mount hyperactive CD4^+^ T-cell responses, leading to active TB.

## Dissection of The Success and Failure of BCG

The success of BCG against childhood TB and its variable efficacy against adult pulmonary disease are explained below by taking into account the heterogeneity of TB, protective versus pathological immunity during *Mtb* infection, and effect of EMb on anti-*Mtb* immune responses.

### Protective Efficacy of BCG Against Childhood TB

As discussed above, the immune system in children is poorly developed with dampened T_H_1 responses and inflammatory pathways (16). Infant CD4^+^ T cells exhibit defective functionality and differentiation bias towards T_H_2 effector cells (17). BCG vaccination alters the immune profile and promotes *Mtb*-specific T_H_1-polarized immune responses in these people (38). Vekemans et al. demonstrated that frequencies of IFN-γ-producing cells and the levels of IFN-γ produced in response to PPD in BCG-vaccinated infants are comparable with those in adults (39). As in the case of adults, CD4^+^ T lymphocytes are the main source of IFN-γ in BCG-vaccinated infants (39). Strong lympho-proliferative response and T_H_1 cytokine secretion in BCG-vaccinated infants have been demonstrated by the other researchers also (40, 41). BCG vaccination has also been shown to induce mycobacterium-specific cytotoxic T lymphocyte responses in neonates (40, 42). Soares et al. demonstrated that CD8^+^ T cells are an important source of IFN-γ, IL-2 and TNF-α in BCG-immunized infants (43). Besides CD4^+^ and CD8^+^ T cells, BCG can also activate unconventional γδ T cells, which play a bridging role between innate and adaptive immunity against *Mtb* and act as an important source of IFN-γ in vaccinated children (44, 45). In contrast to abundant T_H_1 cytokines, BCG-vaccinated newborns demonstrate relatively lower levels of IL-4 and IL-10 (43).

A few studies have also examined the status of immunological memory in BCG-vaccinated infants. It has been observed that newborn BCG vaccination leads to the development of memory CD4^+^ T-cell population with phenotypic characteristics of central memory cells and functional attributes of effector memory cells (46). In a follow-up study, levels of IFN-γ, IFN-γ^+^CD4^+^ T cells, and IFN-γ^+^ γδ T in <1-year-old BCG-vaccinated children were found to be comparable with those present at different time points till ≥5 years (47). Interestingly, Kagina et al. demonstrated a superior CD4^+^ T-cell memory response with BCG administered at 10 weeks (48). Owing to the induction of T_H_1-polarized responses and immunological memory by BCG, vaccinated infants are more likely to respond rapidly and optimally to *Mtb* infection, resulting in the effective containment of the bacilli. Accordingly, BCG confers significant protection against childhood manifestations of TB.

### Variable Efficacy of BCG Against Adult Pulmonary TB

Host response to *Mtb* is more complex in adults. Although a small proportion of TB cases in them can be attributed to hereditary or acquired defects in immune system, a majority of adult pulmonary TB patients demonstrate an aggravated immune response to *Mtb* (23). Maximum burden of adult pulmonary TB lies in the tropics, which account for approximately 95% of TB cases and 98% of TB-related deaths (49). Importantly, people living in the tropics exhibit a stronger tuberculin reaction, compared with those in subtropical and temperate zones.

Higher prevalence of skin test reactivity to PPD-B (*M. avium-intracellulare* antigen) suggests the greater abundance of EMb in the tropics, compared with temperate zones. In the Chingleput trial area, for example, ~90% of 10 to 14-year-old participants exhibited strong reactivity to PPD-B (50). For they carry multiple cross-reactive antigens, EMb can directly modulate the host response to *Mtb*. Accordingly, a more strong response to *Mtb* is observed in the tropics and in elder people, who are more likely to have received greater EMb exposure, compared with younger ones. In the south India trial, 62.0%/48.4% tuberculin positivity (>12 mm with 3 IU of PPD-S) was observed in 15 to 24-year-old male/female participants, which reached 81.8%/64% in 25 to 30-year-old male/female participants, respectively (51). In Karonga prevention study, tuberculin positivity (skin induration of ≥10) was found to increase from ~20% in 20-year-old male participants to 60% in 45-year-old male participants (8). Similar results have been obtained in other studies in tropical Africa (52). On the other hand, no participants in the 5-14 years age group developed grade II or III Heaf reactions (with PPD-S) and only ~15%/~20% of 15 to 24-year-old participants exhibited grade II/grade III Heaf reactions, respectively, in a clinical study in the United Kingdom (53).

It can be inferred that, putatively due to frequent EMb exposure, a large proportion of immunocompetent adults mount a heightened immune response to mycobacterial antigens in the tropics. As an aggravated antimycobacterial immune response leads to TB pathogenesis, many of these people are bound to undergo LTB reactivation into the active disease. Supporting this, people with stronger tuberculin reaction have been found to carry a higher risk of active TB in different clinical trials. Similarly, elevated risk of active TB has been observed in household contacts (of active TB patients), who develop stronger tuberculin reactions (19, 23). Interestingly, male participants have exhibited a more intense tuberculin reaction in clinical studies, which correlates with the higher incidence of adult TB in them (8, 50). It is plausible that frequent EMb exposure and resulting augmentation of antimycobacterial immune response increases the risk of active TB by mechanisms analogous to those in people cured of the previous episode of the disease.

**Figure 1:**
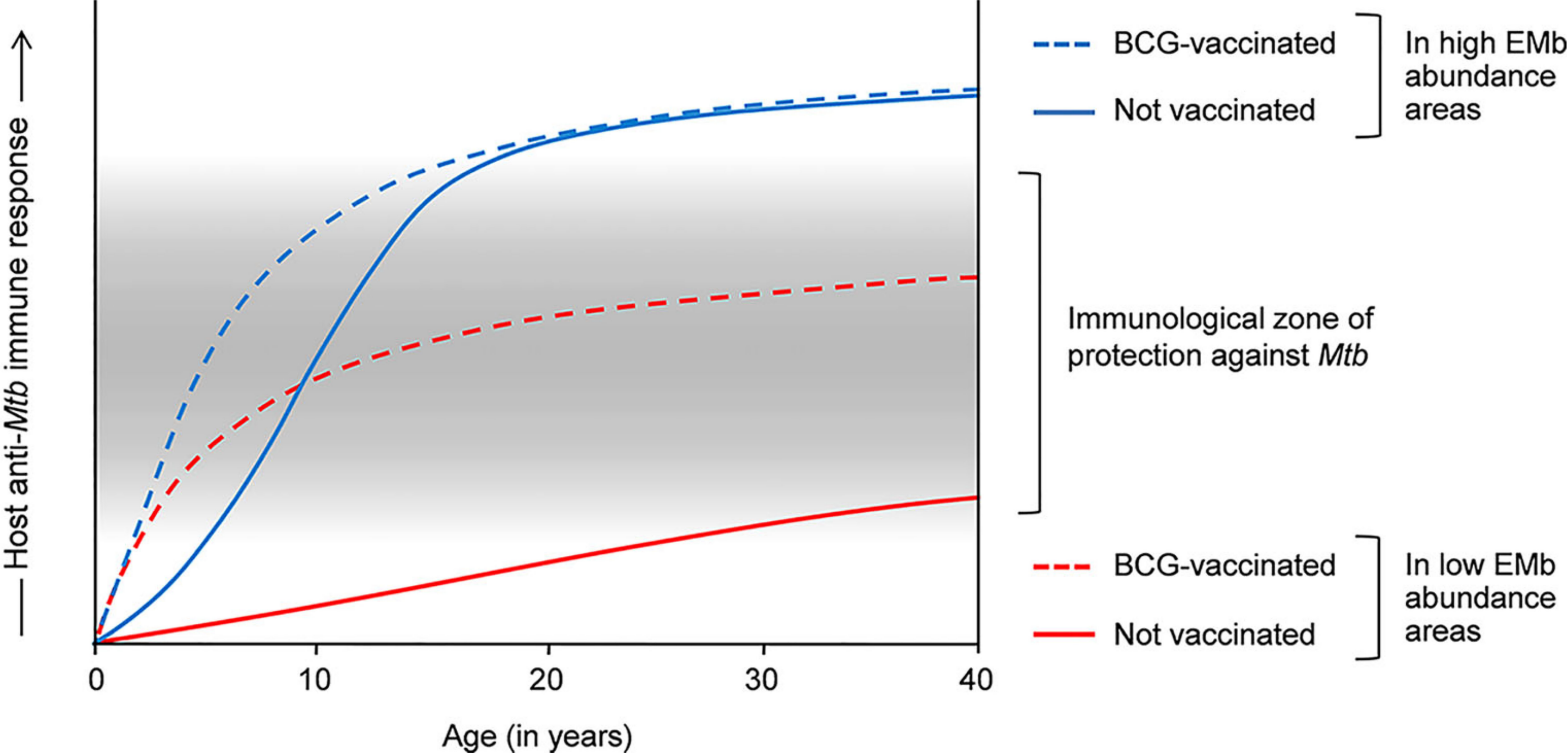
Effects of environmental mycobacteria (EMb) and BCG on the host response to *Mtb* and the cross-talk thereof. Host response to *Mtb* is complex and heterogeneous. Infants and young children have a poorly developed immune system, which is incompetent in containing *Mtb* infection. BCG vaccination in these people promotes T_H_1 responses to *Mtb*, resulting in the effective containment of the bacilli and significant protection against childhood TB (dashed blue and red lines). Owing to the presence of cross-reactive antigens, EMb also activate a degree of immunity against *Mtb* and therefore, confer some protection against childhood TB (blue line). However, frequent EMb exposure leads to the aggravation of anti-*Mtb* immunity in immunocompetent adults, which drives TB pathogenesis and results in higher incidence of pulmonary TB in the tEMb-abundant areas (normal blue line). Similar aggravation of anti-*Mtb* immunity occurs in BCG-vaccinated adults in the EMb-abundant areas and leads to higher incidence of adult pulmonary TB and low efficacy of BCG in these places (dashed blue line). On the other hand, owing to low EMb exposure, BCG-mediated immunity against *Mtb* is not substantially modulated in the adult inhabitants in the areas of lower EMb abundance (dashed red line). Accordingly, vaccinated adults exhibit a moderately intense anti-*Mtb* immune response, which confers significant protection against adult pulmonary TB in these areas.

The host immunological differences also provide an explanation for the variable efficacy of BCG against pulmonary TB in tropics and temperate zones. As most immunocompetent adults would develop a heightened antimycobacterial immune response in the tropics, BCG, which acts by promoting T_H_1-type of anti-*Mtb* immunity, cannot be of much use against adult pulmonary TB in these regions. On the contrary, lesser abundant EMb has a limited effect on antimycobacterial immunity at higher latitudes. Therefore, BCG-mediated immune response persists without significant modulation and vaccinated people exhibit higher protection against adult pulmonary TB in these areas. Supporting this explanation, percentages of tuberculin reactors have been found to be low in the northern temperate countries where BCG confers significant protection, and high in tropical areas where BCG exhibits poor efficacy against adult TB (54). The effects of EMb on anti-*Mtb* immune response and efficacy of BCG are depicted in **Figure 1**. However, it is worth mentioning that factors such as malnourishment and air pollution can also modulate the host antimycobacterial immunity and play a role in defining the vaccine efficacy.

Tuberculin reactions and TB incidence in rural versus urban population also provide firm support to the above explanation for the variable efficacy of BCG against pulmonary TB. Studies have shown that within the same geographical region, higher prevalence of non-specific tuberculin sensitivity is observed in the rural population (55–57). In an only trial of its kind, which compared vaccine efficacy in rural versus urban settings, BCG efficacy was found to be 18% in rural areas, compared with 42% in urban areas (58). In fact, lowest efficacy of BCG against pulmonary TB has been observed in the studies carried out in rural areas (50, 59, 60). It can be deduced that for aggravated antimycobacterial immunity drives pulmonary TB pathogenesis in the tropics, BCG (which acts by inducing anti-*Mtb* immune response) turns out to be ineffective against adult pulmonary TB in these areas.

## Discussion

TB is a heterogeneous disease occurring in different patterns/presentations with distinct mechanisms of pathogenesis (3). In infants and young children, the immune system is poorly developed with dampened T_H_1 responses and inflammatory pathways, rendering them susceptible to *Mtb* (16, 17). BCG vaccination promotes a T_H_1-type of anti-*Mtb* immunity in these people and therefore, confers significant protection against childhood TB (38). In immunocompetent adults, development of pulmonary TB has been attributed to an aggravated anti-*Mtb* immune responses (18, 19). Since a more intense antimycobacterial immune response is observed in the tropics, it is plausible that higher incidence of adult pulmonary TB would be reported from these areas. Also, BCG, which acts by inducing anti-*Mtb* immunity, would not be effective against pulmonary TB in these places. On the contrary, BCG-vaccinated people demonstrate a moderately intense antimycobacterial immune response at higher latitudes. For a moderate antimycobacterial response is protective against *Mtb*, BCG exhibits higher protective efficacy against adult TB in these areas. Firm support for the above explanation of the variable efficacy of BCG is provided by its effectiveness in rural versus urban settings (58). A more intense antimycobacterial immune response is observed in rural areas, wherein the efficacy of BCG has been found to be lower, compared with that in urban places (56, 57).

It can be proposed that a different vaccination approach against adult TB is required in the tropics. Most likely, an effective vaccine against adult TB in these areas would focus on moderating *Mtb*-specific IFN-γ^+^CD4^+^ T-cell responses and balancing pro- and anti-inflammatory pathways. That’s probably how we can prevent adult pulmonary TB and save millions of lives.

## Data Availability Statement

The original contributions presented in the study are included in the article/supplementary material. Further inquiries can be directed to the corresponding author.

## Author Contributions

The author confirms being the sole contributor of this work and has approved it for publication.

## Conflict of Interest

The author declares that the research was conducted in the absence of any commercial or financial relationships that could be construed as a potential conflict of interest.

## Publisher’s Note

All claims expressed in this article are solely those of the authors and do not necessarily represent those of their affiliated organizations, or those of the publisher, the editors and the reviewers. Any product that may be evaluated in this article, or claim that may be made by its manufacturer, is not guaranteed or endorsed by the publisher.

## Abbreviations

TB, tuberculosis; *Mtb*, *Mycobacterium tuberculosis*; BCG, Bacillus Calmette-Guerin; EMb, Environmental mycobacteria; LTB, latent tuberculosis; IFN, interferon.
