# Corrigendum: A Perspective on the Success and Failure of BCG

**DOI:** 10.3389/fimmu.2022.850325

**Published:** 2022-02-09

**Authors:** Pawan Kumar

**Affiliations:** Department of Preventive Oncology, Dr. B. R. Ambedkar Institute Rotary Cancer Hospital, All India Institute of Medical Sciences, New Delhi, India

**Keywords:** tuberculosis, BCG, vaccine efficacy, immune response, environmental mycobacteria, heterogeneity, geographical latitude

In the original article, there was an error in **Figure 1** as published. Specifically, the curve representing the host anti-*Mycobacterium tuberculosis* (Mtb) immune response in unvaccinated people in low environmental mycobacteria (EMb) abundance areas (normal red line) was not drawn correctly. The corrected **Figure 1** appears below.

**Figure 1:**
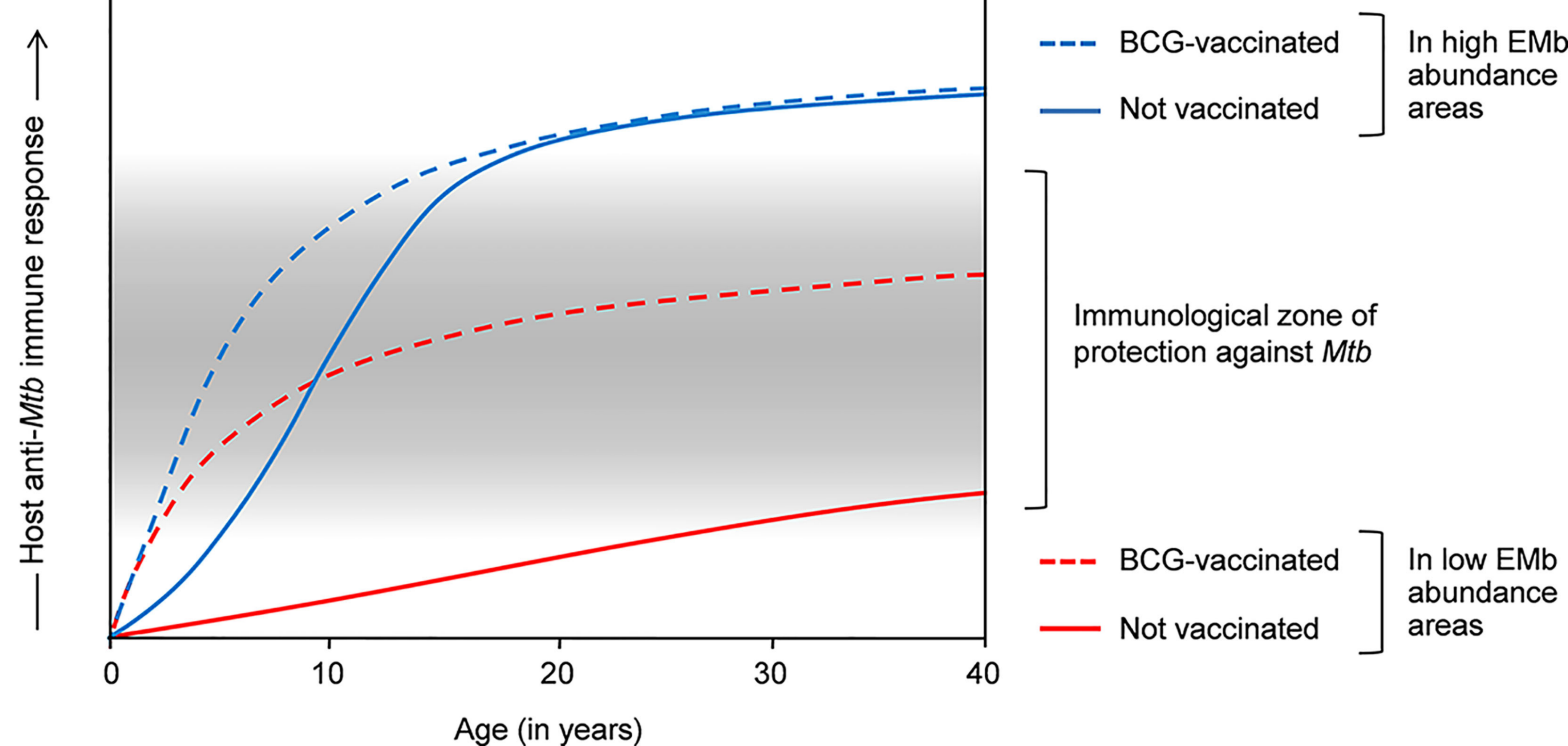
Effects of environmental mycobacteria (EMb) and BCG on the host response to *Mtb* and the cross-talk thereof. Host response to *Mtb* is complex and heterogeneous. Infants and young children have a poorly developed immune system, which is incompetent in containing *Mtb* infection. BCG vaccination in these people promotes T_H_1 responses to *Mtb*, resulting in the effective containment of the bacilli and significant protection against childhood TB (dashed blue and red lines). Owing to the presence of cross-reactive antigens, EMb also activate a degree of immunity against *Mtb* and therefore, confer some protection against childhood TB (blue line). However, frequent EMb exposure leads to the aggravation of anti-*Mtb* immunity in immunocompetent adults, which drives TB pathogenesis and results in higher incidence of pulmonary TB in the EMb-abundant areas (normal blue line). Similar aggravation of anti-*Mtb* immunity occurs in BCG-vaccinated adults in the EMb-abundant areas and leads to higher incidence of adult pulmonary TB and low efficacy of BCG in these places (dashed blue line). On the other hand, owing to low EMb exposure, BCG-mediated immunity against *Mtb* is not substantially modulated in the adult inhabitants in the areas of lower EMb abundance (dashed red line). Accordingly, vaccinated adults exhibit a moderately intense anti-*Mtb* immune response, which confers significant protection against adult pulmonary TB in these areas.

The authors apologize for this error and state that this does not change the scientific conclusions of the article in any way. The original article has been updated.

## Publisher’s Note

All claims expressed in this article are solely those of the authors and do not necessarily represent those of their affiliated organizations, or those of the publisher, the editors and the reviewers. Any product that may be evaluated in this article, or claim that may be made by its manufacturer, is not guaranteed or endorsed by the publisher.

